# Getting to zero: micro-foci of malaria in the Solomon Islands requires stratified control

**DOI:** 10.1186/s12936-021-03779-y

**Published:** 2021-06-05

**Authors:** Tanya L. Russell, Lynn Grignard, Alan Apairamo, Nathan Kama, Albino Bobogare, Chris Drakeley, Thomas R. Burkot

**Affiliations:** 1 Australian Institute of Tropical Health and Medicine, James Cook University, Cairns, Australia; 2 Department of Infection Biology, London School of Hygiene & Tropical Medicine, London, UK; 3 National Vector Borne Disease Control Programme, Ministry of Health and Medical Services, Honiara, Solomon Islands

**Keywords:** *Plasmodium falciparum*, *Plasmodium vivax*, Solomon Islands, Malaria elimination, Heterogeneous transmission

## Abstract

**Background:**

The Solomon Islands has made significant progress in the control of malaria through vector control, access and use of improved diagnostics and therapeutic drugs. As transmission is reduced there is a need to understand variations in transmission risk at the provincial and village levels to stratify control methods.

**Methods:**

A cross-sectional survey of malaria in humans was conducted in the Solomon Islands during April 2018. Nineteen villages across 4 provinces were included. The presence of *Plasmodium* species parasites in blood samples was detected using PCR.

**Results:**

Blood samples were analysed from 1,914 participants. The prevalence of DNA of *Plasmodium falciparum* was 1.2 % (n = 23) and for *Plasmodium vivax* was 1.5 % (n = 28). 22 % (n = 5/23) of *P. falciparum* DNA positive participants were febrile and 17 % of *P. vivax* DNA positive participants (n = 5/28). The prevalence of both *P. falciparum* and *P. vivax* was extremely spatially heterogeneous. For *P. falciparum*, in particular, only 2 small foci of transmission were identified among 19 villages. *Plasmodium falciparum* infections were uniformly distributed across age groups. Insecticide-treated bed net use the night prior to the survey was reported by 63 % of participants and significantly differed by province.

**Conclusions:**

Malaria transmission across the Solomon Islands has become increasingly fragmented, affecting fewer villages and provinces. The majority of infections were afebrile suggesting the need for strong active case detection with radical cure with primaquine for *P. vivax*. Village-level stratification of targeted interventions based on passive and active case detection data could support the progress towards a more cost-effective and successful elimination programme.

**Supplementary Information:**

The online version contains supplementary material available at 10.1186/s12936-021-03779-y.

## Background

Since the beginning of the millennium, substantial progress was made to reduce the global incidence of human malaria, with 12 new countries being certified as malaria-free since 2007 [1, 2]; however, these gains have plateaued over recent years. Countries making significant progress to reduce transmission are committing to malaria elimination by 2030 [3]. As malaria transmission reduces, malaria cases become more spatially heterogeneous [4, 5]. In response, national malaria programmes are encouraged to use local evidence to design and implement a mix of interventions by transmission stratum, rather than utilizing a one-size-fits-all approach [6]. In countries controlling malaria, interventions can be targeted to entire villages with higher malaria incidence until only individual episodes of malaria remain and programmes have the capacity to investigate and respond focally [3].

The Solomon Islands has made significant progress to control malaria through vector control, access and use of improved diagnostics and therapeutic drugs. Vector control has been the principal preventative intervention to reduce malaria transmission over the past two decades with long-lasting insecticidal nets (LLINs) and IRS, both globally and in the Solomon Islands. These strategies have seen *Plasmodium falciparum* replaced by *Plasmodium vivax* as the dominant malaria parasite in the Solomon Islands [7].

Here the annual parasite incidence (API) was reduced from over 200 per thousand in 2003 to just below 30 per thousand in 2014. However, since 2015, the API has increased reaching 107 per thousand in 2019. This resurgence has delayed the goal of malaria elimination with the priority of the 2021–2025 Strategic Plan now seeking to reach zero cases by 2034 [8]. In 2019, four provinces contributed to approximately 86 % of the malaria burden in the country: Central Islands (10.5 %), Guadalcanal (27.1 %), Honiara (15.2 %), and Malaita (33.1 %) [8]. The malaria caseloads, in the other six provinces (Choiseul 0.7 %, Isabel 0.5 %, Makira 4.2 %, Rennell-Bellona 0 %, Temotu 2.5 %, and Western 6.0 %) were responsible for only 14 % of the national malaria burden [8]. This heterogeneity argues for a stratified approach to malaria control. Here, a cross-sectional survey of malaria in humans was undertaken to understand the heterogeneity in malaria transmission risk at the provincial and village levels.

## Methods

### Study site

The cross-sectional study was conducted in the Solomon Islands (− 8.0° S, 157.0° E) in April 2018. The Solomon Islands is hot and wet with an annual rainfall of 2,005 mm (mean for 1999–2017 at Henderson Airport, Guadalcanal Island). Rain falls year round with a peak during January to March. The mean daily coastal temperature ranges between 24 and 30 °C with a mean of 26 °C.

**Fig. 1:**
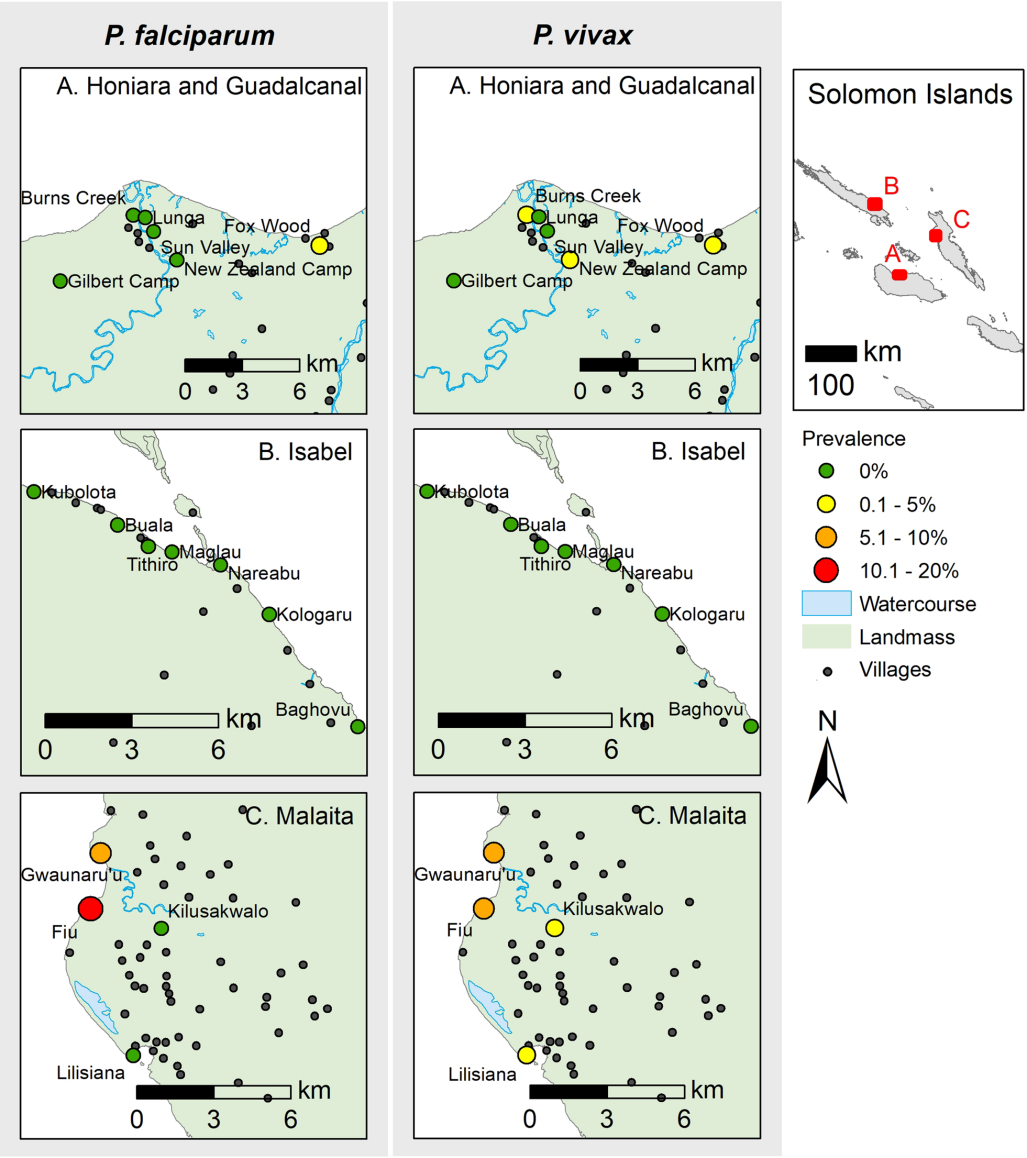
Map of the Solomon Islands (− 8.0° S, 157.0° E) showing villages in the Provinces of: **A** Guadalcanal, **B** Isabel, **C** Malaita and the prevalence of *Plasmodium falciparum* (left) and *Plasmodium vivax* (right). Villages not included in the study are represented as small black circles

The four study sites–Honiara City Council as well as Guadalcanal, Isabel and Malaita Provinces–were selected in consultation with the Ministry of Health and Medical Services, Solomon Islands. Survey sites were selected to cover a range of transmission scenarios from low to high transmission, and to include areas with *P. falciparum* cases based on passive case detection at health facilities. Inclusion criteria required villages to have a minimum resident population of 200, and to be accessible by sea or road. The sites encompassed 19 suburbs and villages (Fig. 1), hereforth termed villages.

### Field procedures

All residents in selected villages, over the age of 5 years, were invited to participate in the study. The only exclusion criteria was the resident’s unwillingness to participate in the study. Residents were equally encouraged to participate across age categories and genders. After enrolment, demographic information and data on possible risk and protective factors associated with mosquito-borne diseases of participants were collected. Data collected included: (1) name, age, gender, household number, (2) fever history, (3) malaria diagnosis and treatment history and (4) access/use of mosquito protection measures. The typanic temperature of participants was measured (Welch Allyn Braun ThermoScan Pro 6000) and any febrile individuals (temperatures > 38 °C) were immediately referred to the nearest health facility.

Each participant provided a ≤ 10 ml blood sample by venepuncture using vacutainers, drawn by a nurse trained and employed by the Ministry of Health and Medical Services. Five µl of selected blood samples was immediately tested for malaria using the AccessBio CareStart rapid diagnostic test (RDT) (G0161) according to the manufacturer’s instructions. Concurrently, 3 × 50 µl blood spots were placed onto cellulose chromatography papers (2 × 7 cm; Whatman® Grade 3MM) and dried under ambient conditions. Dried blood spots were stored in individual snap-lock bags and sealed in an airtight container with silica gel.

Serum was separated by centrifugation at 1,500*g* for 10 min. Serum and clots were initially stored at 4 °C, then frozen at − 20 °C within 4 days, until shipped internationally on dry ice and subsequently stored at − 80 °C until analysed. A unique code was assigned to each participant and their associated samples.

### PCR detection of *Plasmodium spp.* parasites

Dried blood spot (DBS) samples were pooled (4 samples/pool) for extraction using the Chelex/saponin method [adapted from 9]. For each sample, 2 × 3 mm punches were used, being the equivalent of 2 × 2 µl of blood. Each sample was eluted to 150 µl, from which 5 µl of template was used in both nPCR and qPCR. Extracted nucleic acid were amplified in a nested PCR targeting the pan *Plasmodium 18 S* gene [10, 11]. Positive pools were de-pooled and each sample in the pool was individually extracted as above and further analyed by pan genus and species-specific nested PCR [10, 11]. Where all de-pooled samples were negative in the nested PCR, blood clots were analysed by real-time PCR [12] targeting genus-sepcific *18 S Plasmodium* followed by a multiplex reaction targeting *P. falciparum*, *P. vivax*, *Plasmodium malariae* and *Plasmodium ovale*.

### Statistical analysis

The relationship of province on use of insecticide-treated nets (ITNs), other vector control measures, domestic and international travel history and malaria positivity were compared using a chi-squared contingency table (*chisq.test*).

The relationship between malaria PCR positive explanatory variables was analysed using a generalized linear model (GLM; package *MASS*) with a binomial distribution, a binary dependent variable (i.e., negative or positive). Step-forward multi-model inference (MMI), based on ranking the value of the Akaike’s Information Criterion (AIC), was used to select the explanatory variables that best described malaria PCR positivity. The relative strength of evidence for each model within the set of alternatives was assessed using Akaike weights (*w*AIC) where the *w*AIC for each model is interpreted as the probability for the most likely model, with support varying from 0 (no support) to 1 (total support) [13–15]. The most parsimonious model from the final set of nested models was compared with the likelihood ratio test and compared with the *Χ*^2^ distribution [16, 17]. The explanatory variables were village, gender, temperature, age, ITN use and malaria history. Analyses were performed using the R package (v3.5.1).

## Results

### Study population

A total of 1,977 individuals (215 from Honiara, 221 from Guadalcanal, 392 from Western Malaita, and 996 from Isabel) participated in the study. Participants had a median age of 30 years, with 62 % being female (Table 1). The average tympanic temperature of participants during the survey was 37.1 °C, and 34 people had a temperature exceeding 38 °C at the time of the survey, with the maximum temperature recorded being 40.6 °C.

**Table 1:** Study population summary characteristics

Characteristic	Summary
Survey dates	Apr 2018
Number of participants	1,977
Number of samples analysed	1,914
Age–Range	5–86 years
Age–Median	30 years
Percentage female	62 % (n = 1,229)
*P. falciparum* prevalence	0.9 % (n = 17/1914)
*P. vivax* prevalence	1.1 % (n = 22/1914)
Mixed infection prevalence	0.3 % (n = 6/1914)
Percentage *P. vivax*	61 % (n = 28/46)

### Vector control

**Fig. 2:**
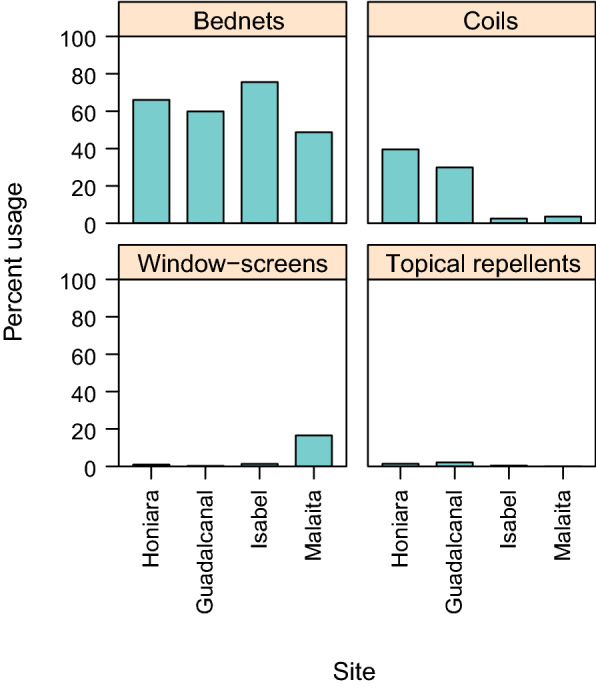
Vector control used by residents of the different provinces

Insecticide-treated bed net use (predominately long-lasting insecticide treated nets (LLINs)) the night prior to the survey was reported by 63 % of participants and significantly differed by province (χ^2^ = 141.49 df = 4, p < 0.0001; Fig. 2), ranging from 48 % in Malaita Province to 75 % in Isabel Province. The use of ITNs also varied by villages from 29 % in Lilisiana, Malaita to 95 % in Hovukoilo Village in Isabel (Additional file 1: Table S1). There was limited use of other mosquito protective measures; 10 % of study participants used mosquito coils (usually a volatile pyrethroid) and 5 % of survey participants’ houses had window screens. Topical repellents were used by only 0.8 % of survey participants. The use of other mosquito control measures was significantly related to the province (χ^2^ = 301.27 df = 4, p < 0.0001), with higher mosquito coil use in Honiara and Guadalcanal (39 and 29 %, respectively), and 16 % of participant houses in Western Malaita having window-screens (Fig. 2).

### *Plasmodium* prevalence by PCR

Blood samples from 1,914 participants were analysed by PCR for *Plasmodium spp.* DNA (henceforth, DNA malaria positive participants are referred to as malaria-positive and used to determine malaria prevalences): 46 participants were malaria positive with 17 individuals positive for *P. falciparum*, 22 with *P. vivax*, 6 with both *P. falciparum* and *P. vivax*, and a single individual with *P. ovale* (Table 2). Of *Plasmodium* PCR positive individuals, seven were febrile (temperature ≥ 38 °C): 2 were positive for *P. falciparum*, 2 were positive for *P. vivax* and 3 were PCR positive with both *P. falciparum* and *P. vivax*; Table 3). Thus, the percentage of febrile or symptomatic participants for *P. falciparum* was 22 % (n = 5/23) and for *P. vivax* was 17 % (n = 5/28).

**Table 2:** The prevalence of *Plasmodium* DNA-positives across provinces in the Solomon Islands among residents of all ages above 5 years

		Number positive				Prevalence		
Province	Participants	Mixed^a^	Pf	Pv	Po	Pf	Pv	Overall
Honiara	211	0	0	4	0	0.0 %	1.9 %	1.9 %
Guadalcanal	369	1	0	3	0	0.3 %	1.1 %	1.1 %
Isabel	946	0	0	0	0	0.0 %	0.0 %	0.0 %
Malaita	388	5	17	15	1	5.7 %	5.2 %	9.8 %
Overall	1,914	6	17	22	1	1.2 %	1.5 %	2.4 %

^a^The mixed infections contained both *P. falciparum* and *P. vivax*

**Table 3:** The number and percentage of participants that were positive for either *P. falciparum*, or *P. vivax* summarized by the various explanatory variables

Parameter	Total	*P. falciparum*		*P. vivax*	
		n	%	n	%
Gender					
Female	1197	14	1.2 %	14	1.2 %
Male	717	9	1.3 %	14	1.9 %
Fever					
Yes	34	5	14.7 %	5	14.7 %
No	1880	18	0.9 %	23	1.2 %
Domestic travel history					
Yes	79	0	0.0 %	2	2.5 %
No	1835	23	1.3 %	26	1.4 %
International travel history					
Yes	49	0	0.0 %	1	2.0 %
No	1865	23	1.2 %	27	1.4 %
Bed net use					
Yes	1275	11	0.9 %	6	0.5 %
No	639	12	1.9 %	22	3.4 %
Malaria history					
Yes	884	14	1.6 %	15	1.7 %
No	1016	9	0.9 %	13	1.3 %
Medicine use					
Yes	33	0	0.0 %	1	3.0 %
No	1881	23	1.2 %	27	1.4 %

**Fig. 3:**
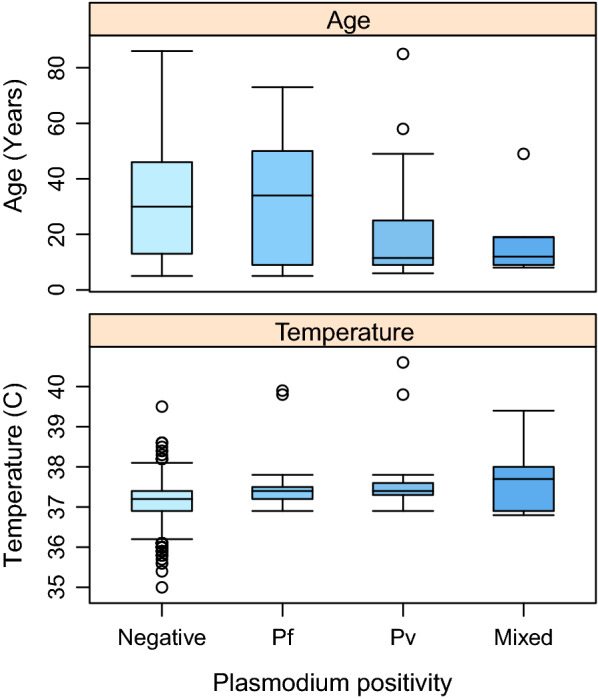
The age and temperature distribution of the survey population by malaria DNA positive status

For *P. falciparum*, the base GLM model was most substantially improved by adding village (100 % *w*AIC support). Sequentially the model was improved by adding temperature (93 % *w*AIC support, Fig. 3). These explanatory variables of village and temperature were significant (log-likelihood ratio test) and were included in the most parsimonious model (Table 4). None of the other remaining candidate factors were able to further improve model fit. Of note is that although the prevalence of *P. falciparum* was reduced almost by half from 1.9 to 0.9 % by using an ITN (Table 3), this factor did not explain sufficient variation to be included in the final model.

**Table 4:** Final set of nested models evaluated to determine which best predicted the prevalence of *Plasmodium* DNA-positives

Model	df	AIC	∆AIC	*w*AIC	χ^2^	*p* value
*Plasmodium falciparum *						
Village	19	183.91	7.31	0.0147		
Village + Temperature	20	176.59	0.00	0.5694	9.31	0.0022*
Village + Temperature + Bed net use	21	177.22	0.63	0.4157	1.37	0.241
*Plasmodium vivax *						
Village	19	253.64	23.87	< 0.0001		
Village + Temperature	20	241.25	11.48	0.0023	14.39	0.0001*
Village + Temperature + Bed net use	21	231.74	1.97	0.2714	11.51	0.0007*
Village + Temperature + Bed net use + Gender	22	229.77	0.00	0.7261	3.98	0.0463*

Model comparison was made on the basis ∆AIC, *w*AIC and goodness-of-fit using maximum likelihood estimation. The full list of explanatory variables included village, gender, temperature, age, bed net use and malaria history

Much of the variation in *P. falciparum* prevalence was explained by village, and this species was extremely heterogeneous across the provinces, with only 2 small foci of transmission identified among 19 villages surveyed (Fig. 1). *Plasmodium falciparum* DNA positive samples were extremely localized, there was 1 mixed positive sample from Guadalcanal (n = 119) and the remaining 22 positive participants were from only 2 villages in Malaita. Overall, 69 % of the *P. falciparum* positives were from a single village, where the prevalence was 15.5 % (Fig. 1). When compared at the provincial level, *P. falciparum* prevalence was highest in Malaita (Table 2).

For *P. vivax*, the base GLM model was most substantially improved by adding village (99 % *w*AIC support). Sequentially the model was improved by adding temperature (87 % *w*AIC support, Fig. 3), bed net use (94 % *w*AIC support) and gender (51 % *w*AIC support). These explanatory variables of village, temperature, bed net use and gender were significant (log-likelihood ratio test), and were included in the most parsimonious model (Table 4). Adding age or malaria history was unable to further improve the model fit. While age was not included in the most parsimonious *P. vivax* model, a univariate GLM did pick up an influence of age on infection (χ^2^ = 9.63, df = 1, p = 0.0019; Fig. 3). For gender, males were more likely to be positive for *P. vivax* (Table 3).

The *P. vivax* positive samples were heterogeneous across the villages, but they were not as extremely localized as the *P. falciparum* infections. *Plasmodium vivax* DNA was detected in participants from Guadalcanal (n = 4), Honiara (n = 4) and Malaita (n = 20), across 7 villages. Overall 63 % of the *P. vivax* positive individuals were found in 2 villages, where the prevalence was 8.0 and 9.7 % (Fig. 1). *Plasmodium spp*. DNA was not detected from Isabel province participants.

## Discussion

Historically in the Solomon Islands, *P. falciparum* was the dominant species with a higher prevalence in younger age groups [18, 19], but as transmission declined, the proportion of *P. vivax* cases increased and the prevalence of sub-microscopic malaria infections was high [20, 21], with malaria transmission becoming increasingly fragmented, affecting fewer villages and provinces [7, 20]. Such heterogeneity in low malaria transmission settings was previously documented [22–25]. In the current study, most infected individuals were afebrile with the prevalence of *P. falciparum* uniformly distributed across age groups. The epidemiology of low malaria transmission observed here is not dissimilar to that encountered at the early 1970 s during the DDT-based Malaria Eradication Programme when prevalence dropped to 1.4 % and *P. vivax* dominated [18]. Similar shifts in malaria epidemiology were also observed in Temotu and Isabel Provinces in the early 2010 s during elimination efforts in these two provinces [26, 27]. In the current study, statistical hotspots were not defined by geospatial statistics due to the difficulty to show statistical significance at such very low transmission intensities where only isolated foci remain [5].

Malaria transmission intensity is a function of both receptivity of the environment to support anopheline vector populations and the presence of malaria parasites in an area or the risk of introduction of malaria parasites [28] with most variability in local exposure to malaria due to differences in mosquito populations densities [29]. Previous work in the Solomon Islands argued that the biting rate of the dominant malaria vector, *Anopheles farauti*, is a better predictor of malaria receptivity than the sporozoite rate or entomological inoculation rate [30]. Considering the fundamental role of mosquito densities and survivorship on malaria transmission, vector control has been directly responsible for large reductions in malaria transmission, not only in the Solomon Islands [31–34], but globally [35]. In the Solomon Islands, household-based LLIN distributions have been continuously implemented since the early 2000 s, with annual indoor residual spraying (IRS) in selected high burden areas until 2015. Here, the impact of LLIN use on the prevalence of *P. falciparum* was not detected, and this is likely a consequence of the low number of infections that were detected and the difficulty associated with detecting statistical significance at low transmission. LLIN use did significantly reduce *P. vivax* transmission with 94 % *w*AIC support.

While malaria transmission has been reduced, it is important to highlight the fragility of these reductions and the ability of malaria transmission to quickly rebound. In the Solomon Islands, malaria incidence rebounded from 20/1,000 people in 1976 to 450/1,000 in 1992 after the Malaria Eradication Programme ended [33]. A recent systematic review across the years 1930–2000 identified 75 malaria resurgences across the globe, with 91 % of resurgences resulting from delays in implementing malaria vector control strategies [36]. Hence, the World Health Organization (WHO) recommends that malaria control efforts, including vector control, must be maintained in receptive areas even after transmission has been eliminated. In the Solomon Islands, malaria transmission across the country has increased during the short time since this survey was completed. The reasons are multi-faceted and may include delays in vector control during decentralisation of the malaria control programme, decreased bioefficacy of LLINs [37], a shortage of LLINs per household, withdrawal of IRS and minimal use of primaquine to treat *P. vivax* hypnozoites [8].

The current WHO recommendations for elimination and post-elimination settings calls for stratified targeting of control efforts by transmission intensity zones [3, 38]. In response, the *Solomon Islands Strategic Plan for Malaria Control and Elimination, 2021–2025* identified 24 high malaria burden health zones by passive case detection in the provinces of Central Islands, Guadalcanal, Honiara, Makira, Malaita, Temotu and Western. This study confirmed the locations of high malaria burden health zones in three of these provinces, defining the residual malaria foci as small in size (e.g., individual villages), and provides evidence supporting the implementation of the strategic plan to target these foci effectively.

The strategic plan outlines the strategies for vector control, case management and surveillance and response. For vector control, the aim is to achieve universal coverage with LLINs, and to use IRS to rapidly reduce incidence in high transmission and outbreak areas. Previous work demonstrated that the risk of being bitten by *An. farauti*, the dominant malaria vector, occurs early in the evening and pre-dominantly in the peri-domestic areas of house verandas and kitchens [39]. Hence, targeted IRS in these high risk structures could enhance malaria control in residual malaria foci [40]. For case management, and in particular primaquine use, the lack of G6PD testing has been a barrier and the aim is to roll out point-of-care G6PD testing to improve usage of primaquine. For surveillance and response, the strategic plan outlines the steps for creating an elimination-ready case-based surveillance system for use in Isabel Province using reactive case detection (RACD) based on the 2-4-7 model. Cases will be reported within two days, investigated within four days and appropriate remedial measure taken within a week. Such stratified malaria control will require improved capacity [41] and data management to rapidly detect and report infection foci as well as to respond with targeted interventions against the vector and parasite [42].

## Conclusions

Pockets of malaria were detected in highly localized foci in the Solomon Islands. This highlights the need for stratified malaria control with increased vector control in high transmission areas and case-based surveillance using RACD in low transmission areas with anti-malarial treatment including radical cure for *P. vivax*. Village-level stratification of targeted interventions could support the progress towards a cost-effective and successful elimination programme.

## Supplementary Information

**Additional file 1: Table S1.** The number and percentage of participants that were positive for either *P. falciparum* or *P. vivax*, summarized by the various explanatory variables.** Figure S1.** Age trends of *Plasmodium* species infections detected by PCR.

## Data Availability

The datasets supporting the conclusions of this article are available in the JCU Tropical Data Hub repository, (10.25903/4054-3m75).

## Abbreviations

ACT: Artemisinin-based combination therapy
AIC: Akaike’s Information Criterion
API: Annual parasite incidence
G6PD: Glucose-6-phosphate dehydrogenase
GLM: Generalized linear model
IRS: Indoor residual spraying
ITN: Insecticide treated net
LLIN: Long-lasting insecticidal net
MDA: Mass drug administration
MMI: Multi-model inference
RACD: Reactive case detection
RDT: Rapid diagnostic test
WHO: World Health Organization
