# Supplementary material for: Getting to zero: micro-foci of malaria in the Solomon Islands requires stratified control
Source: Malar J. 2021 Jun 5;20:248. doi: 10.1186/s12936-021-03779-y (PMC8180101; doi:10.1186/s12936-021-03779-y)
Supplement: Supplementary file 1 — Additional file 1: Table S1. The number and percentage of participants that were positive for either P. falciparum or P. vivax, summarized by the various explanatory variables. Figure S1. Age trends of Plasmodium species infections detected by PCR. [file 12936_2021_3779_MOESM1_ESM.docx]

**This additional file is part of:** Russell TL, Grignard L, Apairamo A, Kama N, Bobogare A, Drakeley C, Burkot TR. Getting to Zero: micro-foci of malaria in the Solomon Islands requires stratified control. *Malaria Journal.*

**Table S1. Insecticide treated bednet use by residents of the different study villages**

| **Province** | **Village** | **N** | **Usage (%)** |
| --- | --- | --- | --- |
|  |  |  |  |
| Honiara | Burns Creek | 116 | 69 |
|  | Gilbert Camp | 99 | 61 |
|  |  |  |  |
| Guadalcanal | Fox Wood | 122 | 65 |
|  | Lunga | 163 | 49 |
|  | New Zealand Camp | 49 | 81 |
|  | Sun Valley | 40 | 57 |
|  |  |  |  |
| Isabel | Baghovu | 100 | 39 |
|  | Buala | 240 | 93 |
|  | Gnulahage | 46 | 89 |
|  | Hovukoilo | 70 | 95 |
|  | Kologaru | 99 | 63 |
|  | Kubolota | 52 | 59 |
|  | Maglau | 115 | 45 |
|  | Nareabu | 134 | 92 |
|  | Tithiro | 140 | 79 |
|  |  |  |  |
| Malaita | Fiu | 104 | 42 |
|  | Gwaunaru’u | 100 | 30 |
|  | Kilusakwalo | 96 | 93 |
|  | Lilisiana | 92 | 29 |

**Figure S1. Age trends of *Plasmodium* species infections detected by PCR.**

Pf = *Plasmodium falciparum,* Pv = *Plasmodium vivax*.

**
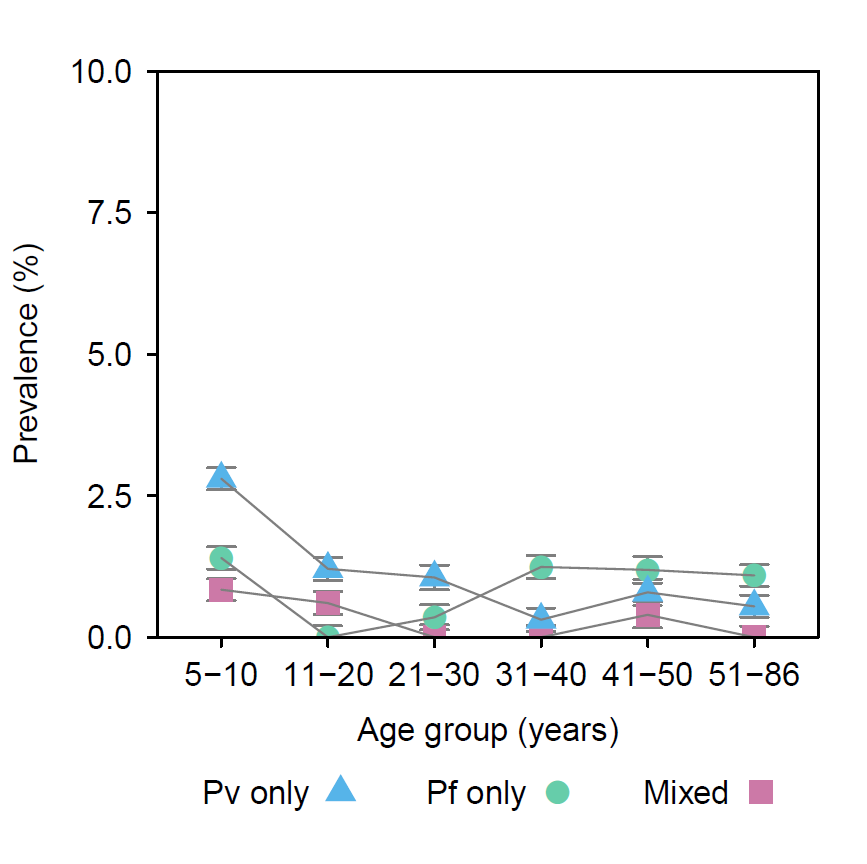
**
